# Impacts of allergic airway inflammation on lung pathology in a mouse model of influenza A virus infection

**DOI:** 10.1371/journal.pone.0173008

**Published:** 2017-02-28

**Authors:** Akira Kawaguchi, Tadaki Suzuki, Yuki Ohara, Kenta Takahashi, Yuko Sato, Akira Ainai, Noriyo Nagata, Masato Tashiro, Hideki Hasegawa

**Affiliations:** 1 Department of Pathology, National Institute of Infectious Diseases, Shinjuku, Tokyo, Japan; 2 Center for Influenza Virus Research, National Institute of Infectious Diseases, Musashimurayama, Tokyo, Japan; Centre National de la Recherche Scientifique, FRANCE

## Abstract

Influenza A virus is the respiratory pathogen responsible for influenza. Infection by the 2009 pandemic influenza A (H1N1) virus caused severe lower airway inflammation and pneumonia. Asthma is a chronic inflammatory disorder of the airways that affects the entire brachial tree, and was one of the commonest underlying medical conditions among patients hospitalized with the 2009 pandemic influenza virus infection. Although respiratory virus infections are the major causes of asthma exacerbation, the mechanism by which influenza exacerbates asthma is poorly understood. Animal models of disease comorbidity are crucial to understanding host-pathogen interactions and elucidating complex pathologies. Existing murine models of influenza virus infection in asthmatics show that asthmatic mice are highly resistant to influenza virus infection, which contradicts clinical observations in humans. Here, we developed a murine model of influenza virus/asthma comorbidity using NC/Nga mice, which are highly sensitive to allergic reactions such as atopic dermatitis and allergic airway inflammation. This model was then used to examine the impact of allergic airway inflammation on lung pathology in the 2009 pandemic influenza virus infected mice. The results showed that induction of acute allergic airway inflammation in pre-existing influenza virus infection had additive effects on exacerbation of lung pathology, which mirrors findings in human epidemiological studies. In contrast, pre-existing allergic airway inflammation protected from subsequent influenza virus infection, which was compatible with those of previous murine models of influenza virus infection in asthmatic mice. These variable outcomes of this murine model indicate that the temporal relation between allergic airway inflammation and influenza virus infection might play a critical role in asthma and influenza comorbidity. Thus, this murine model will further our understanding of how influenza virus infection affects an asthmatic host and may aid the development of strategies to improve treatments and outcomes for asthmatics harboring influenza virus infection.

## Introduction

Influenza A virus is the etiological agent of influenza, which is characterized by sudden onset of high fever and respiratory symptoms such as cough and sore throat, as well as by systemic symptoms such as headache, muscle aches, and fatigue. Influenza viruses continually circulate in human populations worldwide. Annual epidemics result in approximately three to five million cases of severe illness and approximately 250,000 to 500,000 deaths [1]. The influenza pandemic of 2009 was caused by a novel triple reassortant influenza virus, A(H1N1)pdm2009, which first emerged in pigs in Mexico but spread rapidly across the globe [2]. The pandemic influenza occasionally caused severe lower airway inflammation and pneumonia. Among the patients with the 2009 pandemic influenza virus infection, it was noted that asthma was the most common comorbidity among patients hospitalized with influenza during this pandemic [3] [4].

Asthma is a chronic inflammatory airway disorder affecting the entire brachial tree and distressing 300 millions people worldwide, making it a major public health concern [5]. Inhalation of specific allergens or other environmental materials in asthma induces acute airway hypersensitivity, smooth muscle contraction, mucosal edema, and mucus hypersecretion resulting in airway obstruction, which causes symptoms including coughing, wheezing, and chest tightness [6] [7]. Airway obstruction is variable, with symptom-free periods interspersed by periods of repeated exacerbations [8]. Respiratory virus infections are the most common cause of asthma exacerbation [9]. The virus most commonly associated with asthma exacerbation is rhinovirus, followed by coronavirus, respiratory syncytial virus, parainfluenza virus, and influenza virus [10].

Animal models of human infectious diseases have crucial roles in elucidation of the pathogenesis, development of preventions and treatments of the infectious diseases. Similarity between human disease phenotypes and animal model phenotypes is critical factor for elucidation of the pathogenesis of the human diseases. However, susceptibilities of animals to human pathogens differ significantly depending on animal species and strains. Then, phenotypes of infectious diseases on animal model may differ slightly from the human diseases requiring careful interpretation of the animal model phenotypes. Existing murine models of asthma with respiratory virus infection have shown influenza viruses are involved in asthma development [11]. However, other murine models developed to examine the pathogenesis of influenza/allergic asthma show that asthmatic mice are highly resistant to influenza virus infection [12–14], which contradicts the findings of epidemiological studies in humans, implying that further studies using other animal models are needed to elucidate the pathogenesis of this complicated comorbidity of influenza virus infection in asthmatics. An ovalbumin (OVA)-sensitized and OVA-challenged murine model (based on BALB/c mice) is one of the most popular and proven models of allergic airway inflammation [15]. However, it is often difficult to reproduce complicated clinical settings faithfully with animal models using only a few inbred laboratory mice strains because different laboratory strains of mice show different susceptibilities and responses to antigens [16, 17]. An inbred strain of laboratory mice, NC/Nga, has been used as an animal model of human atopic dermatitis [18–20] because they harbor mutations on chromosome 9 that are linked to increased IgE production and increased Th2 responses [21]. Repeated administration of allergens to the skin of these mice induces atopic dermatitis-like skin lesions; this does not happen in BALB/c mice [22, 23]. Moreover, massive and prolonged allergic responses, such as eosinophilic infiltration and IgE production with severe airway inflammation, were observed in OVA-sensitized and OVA-challenged NC/Nga mice, but not in BALB/c mice, indicating that NC/Nga mice are more sensitive to several types of allergic disease/reaction (including allergic airway inflammation) than BALB/c mice [24, 25].

Here, we developed an influenza virus-infection/atopic mouse comorbidity model based on NC/Nga mice and a 2009 pandemic influenza virus, A/Narita/1/2009 (H1N1)pdm09. Using the mouse model, we have investigated the impact of allergen challenges on lung pathology in the pandemic influenza virus infected mice. We observed an acute allergic inflammation in the airways of asthmatic mice soon after influenza virus infection enhanced of pulmonary inflammation, subsequent epithelial goblet cell metaplasia and weight loss without affecting virus replication, raising a hypothesis that the allergen challenges on top of influenza virus infection induced inflammation had additive effects on exacerbation of lung tissue damage. The finding was compatible with those of previous human clinical observations. In contrast, pre-existing allergic airway inflammation inhibited subsequent influenza virus infection, which was consistent with the previous murine models of influenza/allergic asthma. These two different outcomes of this murine model suggest that the temporal relation between influenza virus infection and allergic airway inflammation might be one of the determinants for comorbidity of influenza virus infection in asthmatics. Thus, this murine model will contribute to our understanding of how influenza virus exacerbates and enhances airway pathology in an asthmatic host.

## Materials and methods

### Animals

NC/Nga mice (female, 6 to 8 weeks old) bred under specific pathogen-free conditions were purchased from Japan SLC Inc., Shizuoka, Japan. Animal studies were performed in strict accordance with the Guidelines for Proper Conduct of Animal Experiments of the Scientific Council of Japan. All animal experiments were conducted in strict compliance with animal husbandry and welfare regulations in handled in biosafety level two animal facilities according to the guidelines of the Animal Care and Use Committee of the National Institute of Infectious Diseases, and were approved by this Committee (approval nos. 110032, 112133, 114064, 211055, and 213069). Mice were monitored daily for clinical signs of morbidity and mortality up to 21 days post infection. The human endpoint was used for mice that lost 25% or more of their initial body weight during the study. When the animals met the criteria, they were scored dead and euthanized under excess isoflurane anesthesia according to institutional guidelines.

### Virus

Mouse-adapted influenza virus A/ Narita/1/2009, which was obtained after 15 passages in mice, was used to represent the A(H1N1)pdm09 virus (pH1N1) in this study [26]. The virus was grown in the allantoic cavities of 10-to-11-day-old fertile chicken eggs. Mice were infected via intranasal (IN) administration of 20 μl of PBS containing pH1N1 virus suspension (40,000 plaque-forming units (PFUs)/mouse) into the left nostril.

### Measurement of virus titer

The virus titer was measured according to the method of Tobita et al. [27]. Briefly, 200 μl aliquots of bronchoalveolar lavage fluid (BALF, serially diluted 10-fold) were inoculated into MDCK cells in a six-well plate. After 1 h, each well was washed to remove non-attached virus, and then overlaid with 2 ml of agar medium. The number of plaques was counted by crystal violet staining at 2 days post-inoculation. The virus titer was expressed as pfu/ml.

### Mouse models of allergic airway inflammation

A NC/Nga mouse model of OVA-induced allergic airway inflammation was used in this study, but with slight modification [25, 28, 29]. Six to eight week old female NC/Nga mice were sensitized by two consecutive weekly intraperitoneal (IP) injections of 100 μg of OVA (Sigma-Aldrich, St. Louis, MO) plus 2 mg of alum crystals (InvivoGen, San Diego, CA). One week after the second IP sensitization, the mice were anesthetized by intraperitoneal injection of a mixture of 1.0 mg ketamine and 0.02 mg xylazine in 0.1 ml/10 g body weight and subjected to IN challenge with 20 μl of pH1N1 virus. Control (Mock, non-infected) mice were inoculated with diluted allantoic fluid lacking virus. On the following 2 days, the virus-infected mice were then anesthetized by intraperitoneal injection of a mixture of 1.0 mg ketamine and 0.02 mg xylazine in 0.1 ml/10 g body weight and challenged IN with 100 μg of OVA in 20 μl of PBS. Control mice that did not have allergic airway inflammation were challenged with PBS instead of OVA. Non-infected mice (10 mice per group) and pH1N1-infected mice (20 mice per group) were then monitored for weight loss until 14 days after viral infection. The mice were sacrificed under excess isoflurane anesthesia on 2, 3, or 21 days post-infection and subjected to collection of bronchoalveolar lavage fluid (n = 5 mice per group on 2 and 3 days post-infection) and pathological analysis (n = 3 mice per group on 3 days post-infection, n = 5–11 mice per group on 21 days post-infection). To test the effect of pre-existing acute allergic airway inflammation, 1 week after the second IP sensitization with OVA, the mice were anesthetized by intraperitoneal injection of a mixture of 1.0 mg ketamine and 0.02 mg xylazine in 0.1 ml/10 g body weight and challenged IN with 100 μg of OVA in 20 μl of PBS for 2 consecutive days. Control mice that did not have allergic airway inflammation were challenged with PBS instead of OVA. Then, on the next day after the second IN challenge with OVA, the mice were anesthetized by intraperitoneal injection of a mixture of 1.0 mg ketamine and 0.02 mg xylazine in 0.1 ml/10 g body weight and challenged IN with 20 μl of pH1N1 virus. Control (Mock, non-infected) mice were inoculated with diluted allantoic fluid lacking virus. Mice infected with pH1N1 (n = 10 mice per group) were then monitored for weight loss until 15 days after viral infection. Body weight on Day 0 (immediately before virus infection) was considered to be 100%. Mice were sacrificed under excess isoflurane anesthesia on 0 (immediately before virus infection), 3, or 21 days post-infection and subjected to collection of bronchoalveolar lavage fluid (n = 5 mice per group on 0 and 3 days post-infection) and pathological analysis (n = 3 mice per group on 3 days post-infection, n = 10 mice per group on 21 days post-infection). For the experiments involving neuraminidase inhibitor treatment, 1 week after the second IP sensitization with OVA the mice were anesthetized by intraperitoneal injection of a mixture of 1.0 mg ketamine and 0.02 mg xylazine in 0.1 ml/10 g body weight and challenged IN with 20 μl of pH1N1 virus. On the following 2 days, the virus-infected mice were anesthetized by intraperitoneal injection of a mixture of 1.0 mg ketamine and 0.02 mg xylazine in 0.1 ml/10 g body weight and challenged IN with 100 μg of OVA with or without 800 μg of zanamivir (ZAN) in 50 μl of PBS. The mice were sacrificed under excess isoflurane anesthesia on 3 or 21 days (pH1N1-infected mice) after infection and subjected to collection of bronchoalveolar lavage fluid (n = 5 mice per group on 3 days post-infection) and pathological analysis (n = 3 mice per group on 3 days post-infection, n = 5–10 mice per group on 21 days post-infection). Control (Mock, non-infected) mice were inoculated with diluted allantoic fluid lacking virus. Another group of control mice that did not have allergic airway inflammation were challenged with PBS instead of OVA.

### Collection of bronchoalveolar lavage fluid (BALF)

BALF was harvested by delivering 1 ml of PBS to the lungs through a tracheal cannula. The number and type of cells, virus titer, and levels of cytokines and chemokines in the BALF were then measured. The number of cells in the BALF was counted using Vetscan HM5 (Abaxis Veterinary Diagnostics, Union City, CA). To confirm the morphology of the cells, 50 μl of BALF was centrifuged onto glass slides at 1000 rpm for 10 min using a Shandon cytocentrifuge (Thermo Fisher Scientific Inc., Waltham, MA). The cells were then stained with Giemsa and analyzed by microscopy. The rest of the BALF was centrifuged for 5 min at 500 × g at 4°C to remove cell debris and stored at −80°C until required.

### Analysis of cytokines and chemokines in BALF

The levels of inflammatory cytokines/chemokines in the BALF were measured using the Mouse Cytokine Twenty-Plex Antibody Bead Kit (Invitrogen, San Diego, CA) with 2-fold diluted BALF as an analyte, as described by the manufacturer. These assays can determine the concentrations of the following 20 cytokines and chemokines: granulocyte-macrophage colony-stimulating factor (GM-CSF), gamma interferon (IFN-γ), interleukin 1α (IL-1α), IL-1β, IL-2, IL-4, IL-5, IL-6, IL-10, IL-12, IL-13, IL-17, gamma interferon-induced protein 10 (IP-10/CXCL10), fibroblast growth factor-basic (FGF-Basic), neutrophil-related chemokine KC (KC/GRO), monocyte chemoattractant protein 1 (MCP-1/CCL2), macrophage inflammatory protein 1α (MIP-1α/CCL3), monokine induced by gamma interferon (MIG/CXCL9), tumor necrosis factor alpha (TNF-α), and vascular endothelial growth factor (VEGF). Type I IFNs in BALF were measured using mouse alpha and beta IFN (IFN-α, and -β) enzyme-linked immunosorbent assay (ELISA) kits (PBL Interferon Source, Piscataway, NJ), according to the protocol described by the manufacturer. For experiments involving neuraminidase inhibitor treatment, the mouse CCL2/JE/MCP-1 DuoSet ELISA (R&D Systems Inc., Minneapolis, MN) was used to measure the concentration of MCP-1/CCL2.

### Histopathology and immunohistochemistry

Animals were sacrificed at 3 days or 21 days post-infection. Excised lung tissues were fixed in 10% phosphate-buffered formalin, which was injected into the trachea until the lungs inflated. All the lobes of the fixed lung tissues were then embedded in paraffin, sectioned, and stained with hematoxylin and eosin (H&E) or Periodic acid-Schiff (PAS). The number of PAS positive epithelial cells in lung tissues was obtained by visual estimation over the entire area of the section. Immunohistochemistry was performed with a rabbit polyclonal antibody for type A influenza nucleoprotein (IAV NP) antigen (prepared in our laboratory [30]) as the primary antibody, as previously described [31]. A peroxidase-labeled polymer-conjugated anti-rabbit immunoglobulin (EnVision/HRP, Dako, Glostrup, Denmark) was used as the secondary antibody. Peroxidase activity was detected by diaminobenzidine/hydrogen peroxide.

### Statistical analysis

Statistical analysis was performed using the GraphPad Prism statistical software package (Version 5.0c: Graph Pad Software Inc., CA). Comparisons between two groups were performed using an unpaired student’s t-test or Mann-Whitney U test. Comparisons between more than three groups were performed using one-way ANOVA followed by Tukey’s multiple comparisons test or non-parametric one-way ANOVA (Kruskal-Wallis test) followed by Dunn’s multiple comparison tests. Analyzing data with two independent variables, such as body weight measurements, was performed using two-way ANOVA followed by Sidak’s multiple comparisons test. Data were considered statistically significant at *p*<0.05.

## Results

### Acute allergic airway inflammation exacerbates lung pathology in influenza virus infected mice without affecting pre-existing virus infection

**Fig 1:**
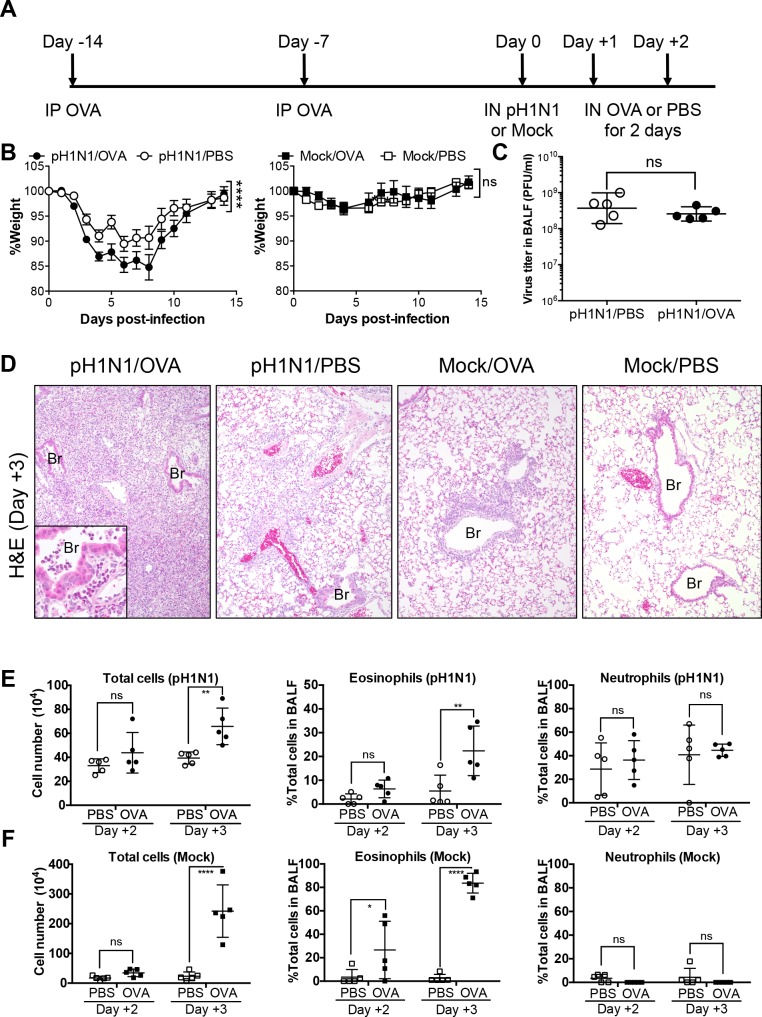
Influenza virus infection and subsequent induction of acute allergic airway responses by OVA in OVA-sensitized NC/Nga mice. (A) Schematic representation of the protocol for OVA sensitization, influenza virus infection, and OVA-induced allergic airway responses. NC/Nga mice were sensitized twice by IP injection of OVA on Day -14 and Day -7 and then infected with a sublethal dose of influenza virus A/H1N1pdm09 (pH1N1) or Mock allantoic fluid (Day 0). On the 2 days following infection, the mice were challenged again with OVA or PBS via the IN route (Day +1 and Day +2). (B) Weight loss was monitored after infection (20 mice in the pH1N1 infection groups and 10 mice in the Mock groups). Error bars represent the mean ± SEM. Weight change data were combined from three (pH1N1 infection groups) or two (Mock groups) independent experiments. (C) On Day 3 post-infection, virus titers in the BALF were measured in a plaque assay (5 mice/group). Data are expressed as scatter plots with the mean viral titer ± SD. (D) Representative lung tissue sections from pH1N1/OVA, pH1N1/PBS, Mock/OVA, and Mock/PBS mice on Day 3 post-infection (3 mice/group). Sections were stained with hematoxylin and eosin (H&E). Original magnification: ×10 or ×40 (inset). (E) Total cell counts and the proportion of inflammatory cells in the BALF form pH1N1/PBS and pH1N1/OVA mice assessed on Day 2 and Day 3 post-infection (5 mice/group). Data are expressed as scatter plots with the mean ± SD. (F) Total cell counts and the proportion of inflammatory cells in the BALF form Mock/PBS and Mock/OVA mice assessed on Day 2 and Day 3 post-infection (5 mice/group). Data are expressed as scatter plots with the mean ± SD. *p<0.05, **p<0.01, ***p<0.001, and ****p<0.0001 (two-way ANOVA or Mann-Whitney U test). IP, intraperitoneal; IN, intranasal; BALF, bronchoalveolar lavage fluid; ns, not significant; Br, Bronchiole. The experiments were repeated independently at least twice.

The first murine model of allergic airway inflammation was set up to examine the effect of a pre-existing influenza virus infection on subsequent allergic airway inflammation. NC/Nga mice were sensitized to OVA (a model allergen) by repeated IP injection with aluminum hydroxide as the adjuvant. The sensitized mice were then infected with a sublethal dose of influenza virus A/H1N1pdm09 (pH1N1). Subsequently, the infected mice were challenged IN with OVA for two consecutive days (Fig 1A). The pH1N1-infected/OVA-challenged mice (pH1N1/OVA) exhibited weight loss much severer than mice not subjected to OVA challenge (pH1N1/PBS); however, non-infected mice with and without OVA challenge (Mock/OVA and Mock/PBS, respectively) did not lose weight (Fig 1B). However, the viral titer in BALF from pH1N1-infected mice was not affected by OVA challenge (Fig 1C). Both allergen challenges and respiratory infections induce pulmonary inflammation, although different types of immune cells take precedence during these reactions. Histopathological analysis revealed that the lung tissues of pH1N1/OVA mice showed massive infiltration of inflammatory cells, including eosinophils (Fig 1D left panel inset), to the bronchioles and alveoli at Day 3 post-infection (Fig 1D left panel). By contrast, the lungs of pH1N1/PBS mice showed limited inflammation of perivascular alveolar areas (Fig 1D, left middle panel). The lungs of Mock/OVA mice also showed mild eosinophilic inflammation around bronchioles while those of Mock/PBS mice remained intact, confirming that acute allergic airway inflammation was induced by the challenges of OVA by IN route for two consecutive days in these mice (Fig 1D, right middle and right panels). Significant increases in total cell numbers and the proportion of eosinophils, which are involved in acute allergic airway inflammation, were observed in the BALF from pH1N1/OVA mice at Day 3 post-infection when compared with those in the BALF from pH1N1/PBS mice (Fig 1E), and also observed in the BALF from Mock/OVA mice when compared with those in the BALF from Mock/PBS mice (Fig 1F) By contrast, the difference between the proportions of neutrophils, which is triggered by influenza virus infection, in these mice was not significant (Fig 1E). Taken together, these observations indicate that OVA-induced acute allergic airway inflammation upon influenza virus infection synergistically exacerbate the lung pathology without affecting influenza virus replication or virus-induced neutrophilic inflammation.

### Influenza virus infection and subsequent acute allergic airway response synergistically enhance chemokine production

**Fig 2:**
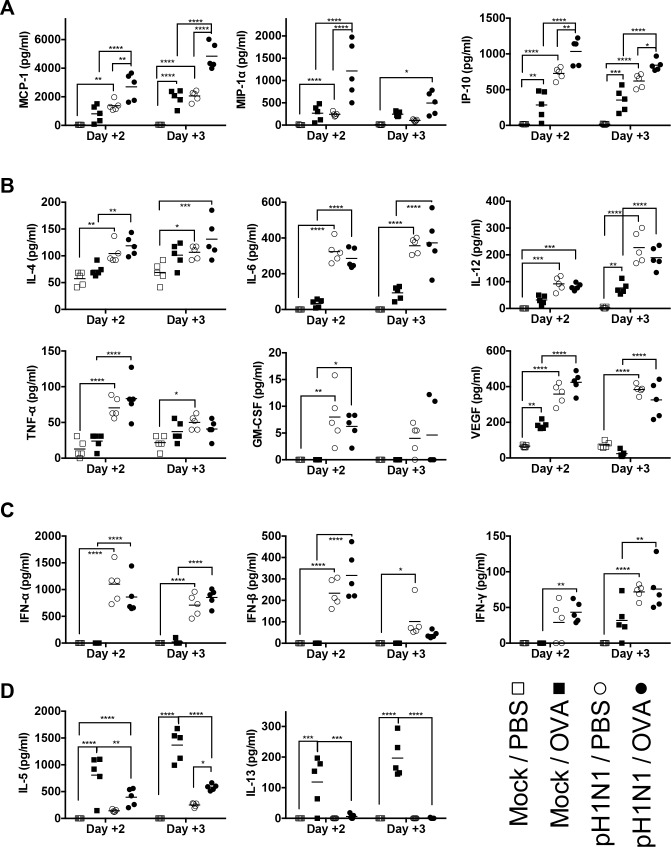
Concentrations of chemokines and cytokines in BALF from mice after influenza virus infection and subsequent induction of allergic airway responses. The concentrations of chemokines and cytokines in BALF from pH1N1/OVA, pH1N1/PBS, Mock/OVA, and Mock/PBS mice were measured on Day 2 and 3 post-infection (5 mice/group). (A) Concentrations of MCP-1/CCL2, MIP-1α/CCL3, and IP-10/CXCL10 in BALF. (B) Concentrations of cytokines, IL-4, IL-6, IL-12, TNF-α, GM-CSF, and VEGF, in BALF. (C) Concentrations of IFN-α, IFN-β, and IFN-γ in BALF. (D) Concentrations of IL-5 and IL-13 in BALF. Data are expressed as scatter plots with the mean. *p<0.05, **p<0.01, ***p<0.001, and ****p<0.0001 (two-way ANOVA). The experiments were repeated twice independently.

As cytokines and chemokines induced by virus infection or allergic response are crucial molecules that regulate inflammation processes in the lung, and because their concentrations correlate with the severity of illness, we measured the amounts of various cytokines and chemokines in the BALF of mice. We found that expressions of MCP-1/CCL2, MIP-1α/CCL3, and IP-10/CXCL10 were significantly higher in pH1N1/PBS and Mock/OVA mice than in Mock/PBS mice. Furthermore, the levels of these three chemokines in pH1N1/OVA mice were markedly higher than those in the other three groups (Fig 2A). In contrast, the levels of IL-4, IL-6, IL-12, TNF-α, GM-CSF, and VEGF (Fig 2B), and those of IFN-α, IFN-β, and IFN-γ (Fig 2C), in pH1N1/OVA mice were not higher than those in pH1N1/mock mice while the levels of these cytokines and interferons in pH1N1/PBS mice were higher than those in Mock/PBS mice (Fig 2B and 2C). These observations suggested that acute allergic immune responses in the airways soon after influenza virus infection had synergistic effects of productions of MCP-1/CCL2, MIP-1α/CCL3, and IP-10/CXCL10 with influenza virus infection induced immune responses and that these chemokines might play a pivotal role in exacerbating the pulmonary inflammation in influenza virus infected mice with subsequent allergen challenges. By contrast, although the levels of IL-5 and IL-13 were significantly higher in Mock/OVA mice than in Mock/PBS mice, they were markedly lower in pH1N1/OVA mice than in Mock/OVA mice (Fig 2D), confirming that the exacerbation of the lung pathology observed in this model might be attributable to synergistic increase of chemokines production induced by both of pre-existing influenza virus infection and subsequent allergic airway inflammation and not to deterioration of acute allergic airway inflammation which is a Th2-driven inflammatory response.

### Pre-existing acute allergic airway inflammation protects from subsequent influenza virus infection and reduces virus-induced cytokine and chemokine production

**Fig 3:**
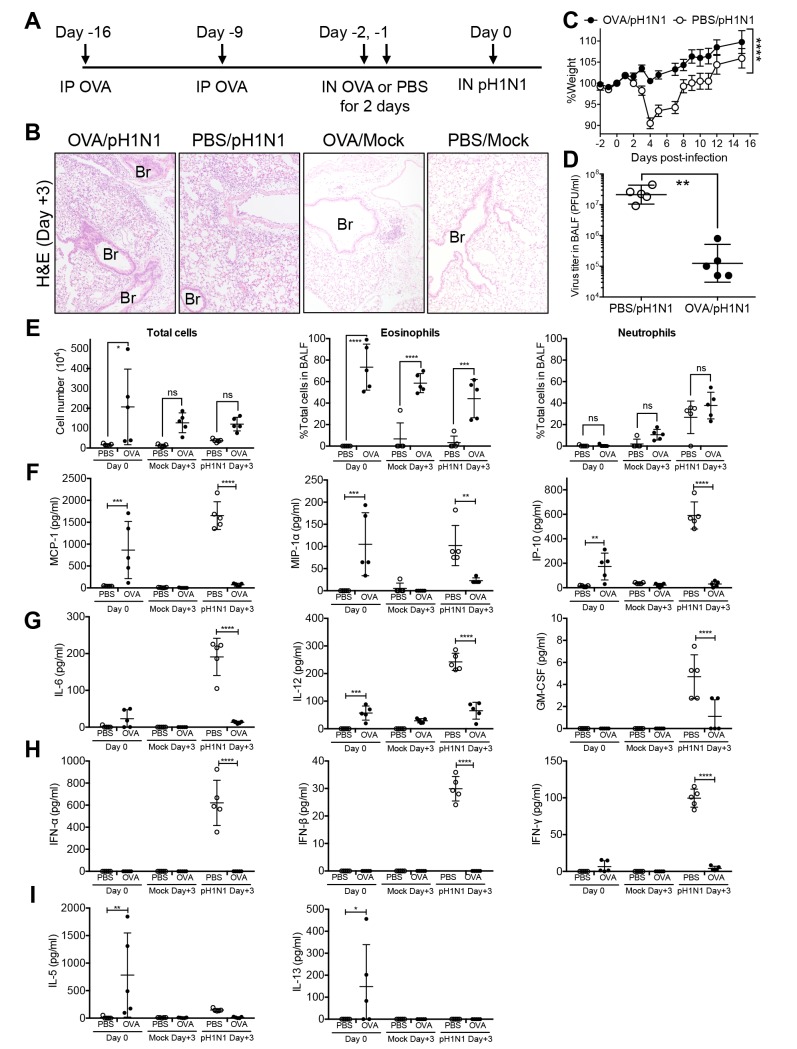
Influenza virus infection of OVA-challenged NC/Nga mice with pre-existing acute allergic airway inflammation. (A) Schematic showing OVA sensitization, OVA-induced allergic airway inflammation, and influenza virus infection. NC/Nga mice were sensitized twice (via the IP route) with OVA (Day -16 and Day -9) and then subsequently challenged IN with OVA on two consecutive days (Day -2 and Day -1). On the day following the final OVA challenge (Day 0), the mice were inoculated IN with a sublethal dose of influenza virus A/H1N1pdm09 (pH1N1). (B) Representative lung tissue sections from OVA/pH1N1, PBS/pH1N1, OVA/Mock, and PBS/Mock mice on Day 3 post-infection (3 mice/group). Hematoxylin and eosin (H&E) staining. Original magnification: ×10. (C) Weight loss was monitored after infection (10 mice/group). Error bars represent the mean ± SEM. Weight change data were combined from two independent experiments. (D) On Day 3 post-infection, virus titers in the BALF were measured in a plaque assay (5 mice/group). Data are expressed as scatter plots with the mean viral titer ± SD. (E) Total cell counts and the proportion of inflammatory cells in the BALF assessed on Day 0 and Day 3 post-infection (5 mice/group). Data are expressed as scatter plots with the mean ± SD. (F to I) Concentrations of chemokines and cytokines, and interferons in BALF from influenza virus-infected mice with pre-existing allergic airway inflammation. The concentrations of chemokines (F), proinflammatory cytokines (G), interferons (H), Th2 cytokines (I) in BALF from PBS/Mock, OVA/Mock, PBS/pH1N1 and OVA/pH1N1 mice were measured before (Day 0) and after (Day 3) infection (5 mice/group). Data are expressed as scatter plots with the mean ± SD. *p<0.05, **p<0.01, ***p<0.001, and ****p<0.0001 (two-way ANOVA or Mann-Whitney U test). Br, Bronchiole. The experiments were repeated independently at least twice.

Recent reports showed that acute allergic airway inflammation accelerates the clearance of influenza virus [12–14], which appears to contradict our present observations. To explore the reasons for these apparent differences, we modified the experimental protocol by reversing the order of the interventions. NC/Nga mice were first sensitized with OVA by repeated IP injection of OVA with aluminum hydroxide as the adjuvant. Then, the sensitized mice were challenged IN with OVA for two consecutive days. On the day of the last OVA challenge, the mice were infected with a sublethal dose of pH1N1 (Fig 3A). Histopathological analysis of the lungs from the pH1N1-infected mice that were pre-challenged with OVA (OVA/pH1N1) revealed that inflammation was limited and similar to that in the lungs from the pH1N1-infected mice that were not pre-challenged with OVA prior to viral infection (PBS/pH1N1) at Day 3 post-infection (Fig 3B left and left middle panels). The lungs of non-infected mice with OVA challenge (OVA/Mock) showed subtle eosinophilic infiltrations around bronchioles, confirming that the allergen challenges in this protocol induced acute allergic airway inflammation (Fig 3B, right middle panel). Interestingly, OVA/pH1N1 mice did not show weight loss while PBS/pH1N1 mice suffered significant weight loss (Fig 3C). We also observed significant reductions in the viral titer in the BALF from OVA/pH1N1 mice (Fig 3D). The proportions of eosinophils, which are involved in acute allergic airway inflammation, were higher in BALF from mice with OVA challenge (OVA/Mock or OVA/pH1N1) than in those from mice without OVA challenge (PBS/Mock or PBS/pH1N1) regardless of influenza virus infection before (Day 0) and after (Day 3) infection. In addition, total cell numbers in the BALF from mice with OVA challenge were higher than those in the BALF from mice without OVA challenge before infection (Day 0), whereas the differences between the numbers in these mice were not significant regardless of influenza virus infection at Day 3 (Fig 3E). A significant increase in the proportion of neutrophils, which is triggered by influenza virus infection, was observed in the BALF from both OVA/pH1N1 mice or PBS/pH1N1 mice at Day 3 post-infection when compared with those in the BALF from mice without infection (OVA/Mock or PBS/Mock, respectively) regardless of OVA challenge (*p*<0.001, OVA/pH1N1 vs. OVA/Mock; *p*<0.001, PBS/pH1N1 vs. PBS/Mock). However, the difference between the proportions of neutrophils in OVA/pH1N1 and PBS/pH1N1 mice was not significant at Day 3 post-infection (Fig 3E). Taken together, these observations indicated that pre-existing OVA-induced acute allergic airway inflammation inhibited influenza virus replication without affecting virus-induced inflammatory cells responses in the early phase of infection. Then, we measured the amounts of proinflammatory cytokines and chemokines in the BALF of mice. Before infection (Day 0), MCP-1/CCL2, MIP-1α/CCL3, and IP-10/CXCL10 levels were higher in OVA challenged mice (OVA) than those in mice without OVA challenge (PBS) (Fig 3F), whereas the levels of these chemokines in OVA/pH1N1 mice fell by Day 3 post-virus infection and the levels in PBS/pH1N1 were higher than those in OVA/pH1N1 mice (Fig 3F). In addition, these chemokine levels in mice without virus infection at Day 3 were diminished. At Day 3 post-infection, expression of IL-6, IL-12, GM-CSF, IFN-α, IFN-β, and IFN-γ was also significantly higher in mice not pre-challenged with OVA (PBS/pH1N1) than in mice that were pre-challenged (Fig 3G and 3H). Meanwhile, the levels of IL-5 and IL-13, which are Th2 cytokines, were significantly higher in the BALF of OVA mice than those of PBS mice before infection (Day 0) although they declined after infection (Day 3) regardless of virus infection (Fig 3I). Taken together, these results suggested that a pre-existing acute allergic immune response inhibited the replication of influenza virus, decreased virus-induced cytokine and chemokine production, and reduced the severity of influenza virus-induced illness, which is compatible with previous findings [12–14].

### Pre-existing acute allergic airway inflammation suppresses influenza virus infection of the bronchiolar epithelium

**Fig 4:**
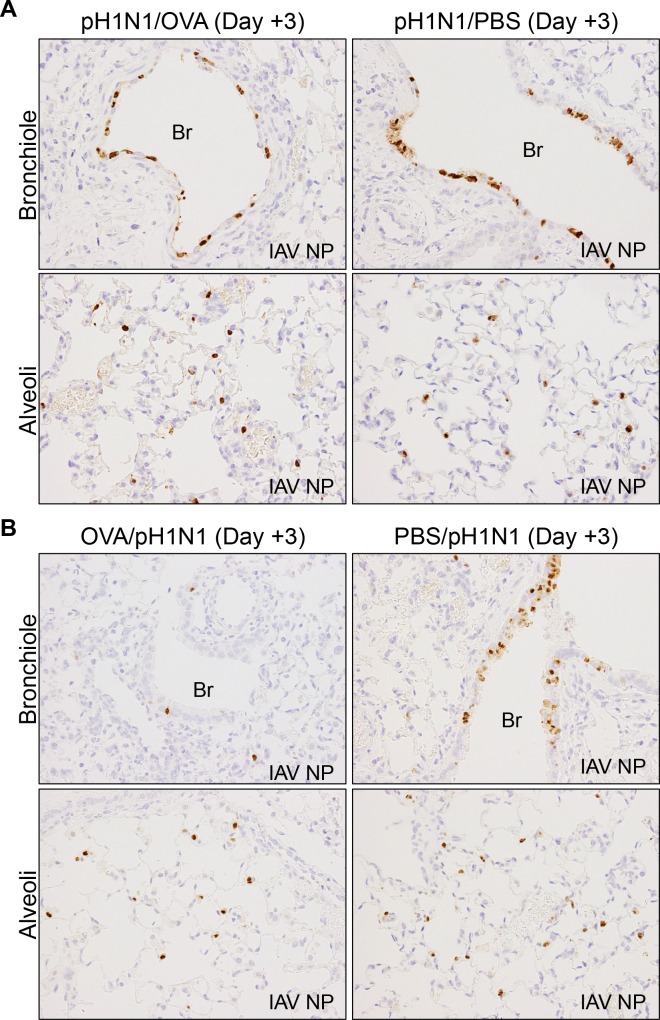
Immunohistochemical analysis of influenza A virus NP antigen (IAV NP) expression in the bronchiolar and alveoli epithelium of pH1N1-infected mice at Day 3 post-infection. (A) Representative sections of lung tissue from mice infected with influenza virus followed by induction of allergic airway responses using OVA (pH1N1/OVA) or from mice infected with influenza and subsequently challenged with PBS (pH1N1/PBS). (B) Representative sections of lung tissue from the mice infected with influenza virus and pre-existing OVA-induced s inflammation (OVA/pH1N1) and from mice infected with influenza virus in the absence of allergic airway inflammation (PBS/pH1N1) The data are representative of 3 mice/group from two independent experiments. Original magnification: ×40. Br, Bronchiole.

The two murine models described above (i.e., the effect of allergic airway responses on pre-existing influenza virus infection (Figs 1 and 2) and the effect of influenza virus infection on pre-existing acute allergic airway inflammation (Fig 3)), show conflicting results with respect to the relationship between acute allergic airway inflammation and influenza virus infection. To address the reasons for this discrepancy, we performed immunohistochemical analysis of lung tissues using an anti-influenza NP antibody to examine the distribution of viral infected cells. We detected similar levels of influenza virus antigens in both the bronchiolar and alveolar epithelium of pH1N1/OVA and pH1N1/PBS mice at Day 3 post-infection, irrespective of subsequent OVA challenge (Fig 4A). By contrast, compared to mice not pre-challenged with OVA (PBS/pH1N1), we observed a marked reduction in the amount of influenza virus antigen in the bronchiolar epithelium of mice that were pre-challenged with OVA (OVA/pH1N1), despite the presence of abundant infected cells in the alveolar epithelium (Fig 4B). These observations indicated that pre-existing acute allergic airway inflammation suppressed the influenza virus replication specifically in the bronchiolar airway epithelium (the site affected by asthma and allergic airway inflammation) but not in the alveolar epithelium (which is unaffected by asthma and allergic airway inflammation); thus pre-existing acute allergic airway inflammation decreased the level of virus-induced cytokine and chemokine production, and then reduced the severity of influenza virus-induced illness. On the other hand, subsequent allergic airway inflammation in influenza virus infected lung appears to have a negligible effect on viral replication in the bronchiolar epithelium, which triggers excessive chemokine production and exacerbates lung pathology.

### Influenza virus infection and subsequent induction of acute allergic airway inflammation promote goblet cell metaplasia of the bronchiolar epithelium

**Fig 5:**
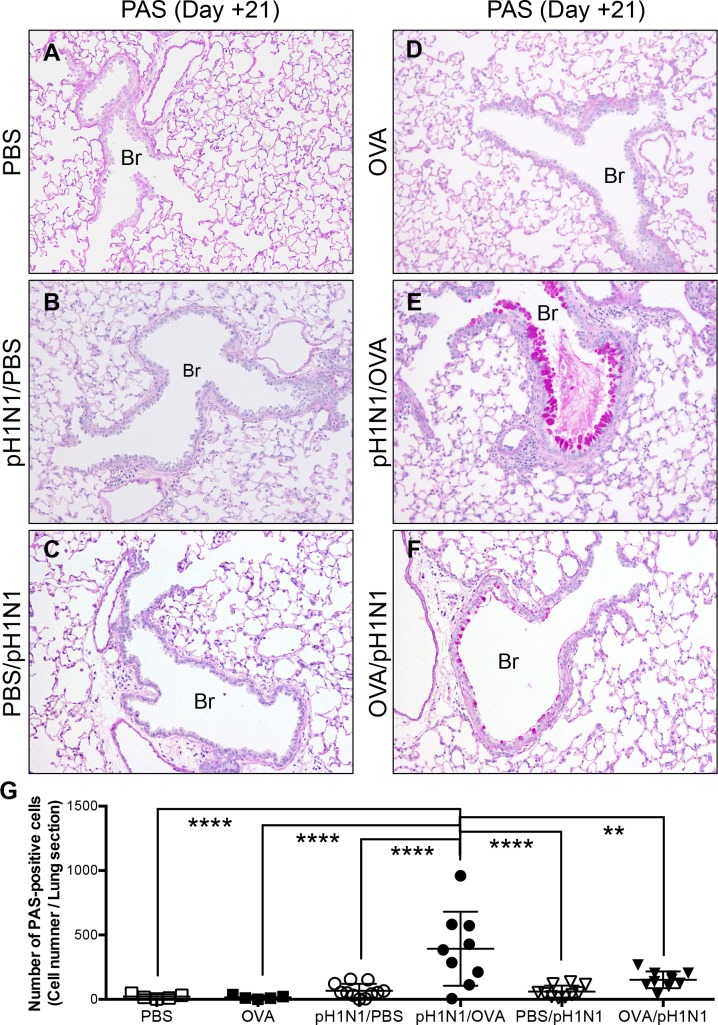
Histopathological analysis for bronchiolar epithelial goblet cell metaplasia at Day 21 post-infection. Representative PAS-stained sections of lung tissues from (A) control mice (inoculated with PBS alone), (B) mice inoculated with influenza virus followed by PBS (pH1N1/PBS), (C) mice inoculated with PBS followed by influenza viral infection (PBS/pH1N1), (D) mice challenged with OVA but not infected with influenza virus (OVA), (E) mice infected with influenza virus followed by OVA challenge (pH1N1/OVA), and (F) mice challenged with OVA and then infected with influenza virus (OVA/pH1N1). The data are representative of 5 to 11 mice/group from three independent experiments. Original magnification: 20×. Br, Bronchiole. (G) Numbers of PAS positive epithelial cells in lung tissues from PBS, OVA, pH1N1/PBS, pH1N1/OVA, PBS/pH1N1, and OVA/pH1N1 were obtained by visual estimation over the entire area of the section. The data are expressed as scatter plots with the mean ± SD of results for 5 to 11 mice/group from three independent experiments. **p<0.01, and ****p<0.0001 (one-way ANOVA).

Asthma is characterized by chronic inflammation of the bronchioles, which causes breathing difficulties and is associated with airway remodeling (alteration of the normal architecture of the bronchiolar walls, including epithelial goblet cell metaplasia, subepithelial fibrosis and thickening of the bronchial smooth muscle wall) [7]. Histopathological analysis of lung tissues revealed that the bronchiolar epithelium of control mice was ciliated and cuboidal, and was not stained by Periodic acid-Schiff (PAS) (Fig 5A; PBS). The same was true for pH1N1-infected mice not challenged with OVA at Day 21 post-infection (Fig 5B and 5C; pH1N1/PBS and PBS/pH1N1). OVA-challenged mice not infected with pH1N1 (Fig 5D; OVA) also showed normal bronchiolar architectures without epithelial goblet cell metaplasia indicating that OVA challenge for two consecutive days in these murine models was not sufficient to induce chronic allergic airway reactions such as the airway remodeling. However, numerous PAS-positive epithelial cells were observed in the bronchioles of pH1N1-infecte mice with subsequent OVA-challenged mice at Day 21 post-infection (Fig 5E and 5G; pH1N1/OVA) despite the absent of obvious structural changes in subepithelial regions, suggesting that influenza virus infection and subsequent induction of acute allergic airway inflammation promoted bronchiolar epithelial goblet cell metaplasia. A few PAS-positive epithelial cells were also observed in the bronchioles of pH1N1-infected mice with pre-existing allergic airway inflammation (OVA/pH1N1), though the difference of the number of PAS-positive cells compared to that of PBS/pH1N1 mice was not statistically significant. In addition, the number of PAS-positive cells in the bronchiolar epithelium of OVA/pH1N1 mice (which showed low levels of virus infection) was significantly lower than that in pH1N1/OVA mice (Fig 5F and 5G). Taken together, these observations indicated that influenza virus infection of the bronchiolar epithelium in mice with acute allergic airway inflammation had promoted goblet cell metaplasia of the airway epithelium without affecting the subepithelial region of airway accordance with the viral load.

### Treatment with a neuraminidase inhibitor reduces influenza virus-induced inflammatory responses, but does not inhibit infection of the bronchiolar epithelium or subsequent epithelial metaplasia in influenza virus-infected/OVA-challenged mice

**Fig 6:**
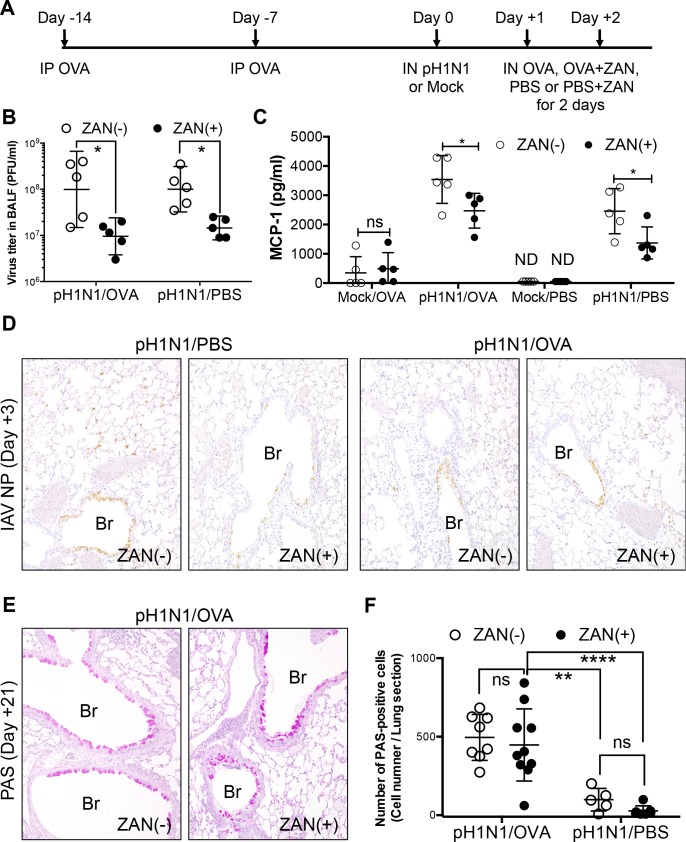
Treatment of influenza virus-infected/OVA-challenged NC/Nga mice with a neuraminidase inhibitor, zanamivir (ZAN). (A) Schematic showing OVA sensitization, influenza virus infection, OVA-induced allergic airway responses, and ZAN treatment. NC/Nga mice were sensitized twice (via the IP route) with OVA (Day -14 and Day -7) and then infected with a sublethal dose of influenza virus A/H1N1pdm09 (pH1N1) or Mock infected with allantoic fluid (Day 0). On the day following infection, mice were administered OVA or PBS with or without 800 μg of ZAN (via the IN route) for two consecutive days (Day +1 and Day +2). (B) On Day 3 post-infection, virus titers in the BALF were measured in a plaque assay (5 mice/group). Data are expressed as scatter plots with the mean viral titer ± SD. *p<0.05 (one-way ANOVA). The experiments were repeated twice independently. (C) Concentration of MCP-1/CCL2 in the BALF measured on Day 3 post-infection (5 mice/group). Data are expressed as scatter plots with the mean ± SD. *p<0.05 (two-way ANOVA). The experiments were repeated twice independently. (D) Representative sections of lung tissue obtained from pH1N1-infected/OVA-challenged or pH1N1-infected/PBS-challenged mice (treated or not with ZAN) on Day 3 post-infection and stained for influenza A virus NP antigen (IAV NP). The data are representative of 3 mice/group from two independent experiments. Original magnification: 20×. (E) Representative PAS-stained sections of lung tissue from pH1N1-infected/OVA-challenged mice (treated or not with ZAN) obtained on Day 21 post-infection. The Data are representative of 5 to 10 mice/group from two independent experiments. Original magnification: 20×. Br, Bronchiole. (F) Numbers of PAS positive epithelial cells in lung tissues from pH1N1-infected/OVA-challenged or pH1N1-infected/PBS-challenged mice (treated or not with ZAN) on Day 3 post-infection were obtained by visual estimation over the entire area of the section. The data are expressed as scatter plots with the mean ± SD of results for 5 to 10 mice/group from two independent experiments. **p <0.01, and ****p<0.0001 (two-way ANOVA).

Neuraminidase inhibitors suppress the multiple cycles of replication of influenza A and B viruses and are commonly used to treat influenza in clinical practice. We examined the effects of an inhaled neuraminidase inhibitor on influenza virus-infected/OVA-challenged mice. To do this, we treated mice with zanamivir (ZAN) via the IN route at the same time as they were challenged with OVA. This was repeated on two consecutive days post-influenza virus infection (Fig 6A). We noted a significant reduction in the viral titer in the BALF from ZAN-treated (ZAN (+)) mice on Day 3 post-infection compared with those in the BALF of mice without ZAN treatment (ZAN (-)), irrespective of OVA challenge (pH1N1/OVA or pH1N1/PBS) (Fig 6B). In addition, the levels of MCP-1/CCL2 in the influenza virus-infected (pH1N1/OVA or pH1N1/PBS) ZAN-treated mice on Day 3 post-infection were significantly lower than those in infected mice that were not treated with ZAN, again, irrespective of OVA challenge; however, levels in OVA-challenged mice not infected with influenza virus (Mock/OVA) were not affected by ZAN treatment (Fig 6C). The results suggested that, although ZAN treatment reduced influenza virus-induced inflammatory responses by inhibiting viral replication, it had no effect on acute allergic airway inflammation. Immunohistochemical analysis revealed viral antigens in the bronchiolar epithelium of influenza virus-infected mice regardless of ZAN treatment (pH1N1/PBS+ZAN, pH1N1/PBS, pH1N1/OVA+ZAN and pH1N1/OVA) at Day 3 post-infection (Fig 6D), indicating that ZAN treatment in this murine model had a negligible impact on influenza virus infection of the bronchiolar epithelium. In addition, PAS staining of mice at Day 21 post-infection revealed abundant goblet cells in the bronchiolar epithelium of influenza virus-infected/OVA-challenged mice regardless of ZAN treatment (Fig 6E and 6F). These observations suggested that a mild reduction in influenza virus replication by treatment with antiviral drugs, such as neuraminidase inhibitors, is not sufficient to prevent bronchiolar epithelial metaplasia which is induced by influenza virus infection and followed by induction of allergic airway inflammation, and that impact of allergic airway inflammation upon influenza virus-infected bronchiolar epithelium might play a pivotal role in subsequent epithelial metaplasia in the bronchioles in influenza virus-infected/OVA-challenged mice.

## Discussion

Asthma is a complex disease caused by multiple genetic and environmental parameters, and is closely associated with respiratory virus infections [9]. Clinical and epidemiological data demonstrate that people with asthma who become infected with influenza virus are at high risk of hospital admission [3, 4]. Elucidation of the pathogenesis of these complicated comorbidities of infectious diseases and autoimmune disorders is important for development of better therapeutics and prevention. Animal models of human infectious diseases have crucial roles in elucidation of the pathogenesis of the diseases, and similarity between human disease phenotypes and animal model phenotypes is critical factor for elucidation of the pathogenesis of the diseases. However, susceptibilities of animals to human pathogens differ significantly depending on animal species and strains. Then, phenotypes of infectious diseases on animal model may differ slightly from the human diseases requiring careful interpretation of the animal model phenotypes.

Existing murine models of asthma/influenza virus comorbidity show seemingly contradictory findings [11–14, 32–34]. Some models demonstrate that influenza virus infection plays a role in inducing asthma development and increases airway sensitization during the acute phase of infection; however, it does not lead to a significant deterioration in the animals’ condition [32, 34]. Other models show that acute allergic airways are resistant to influenza virus infection [12–14] and that influenza virus hinders the recruitment of eosinophils and Th2 cells to the airways after allergen challenge [33]. Influenza virus infection induces Th1 responses, which in turn inhibit the development of Th2 cells, suggesting that influenza virus infection and asthmatic reactions have opposing effects on the immune response. These observations appear to be inconsistent with epidemiological findings that asthma is a significant risk factor for influenza virus infection [3, 4]. This contradiction prompted us to examine how asthma affects influenza virus infection using a novel model based on NC/Nga mice.

Here, we showed that asthmatic mice suffer more severe lung pathology with acute allergic airway inflammation after influenza virus infection. Interestingly, there was no correlation between exacerbation of lung pathology and viral burden. Influenza virus infection injures the host through viral replication-mediated tissue damage and by inducing a harmful inflammatory response. The absence of a correlation between viral burden and exacerbation of lung pathology in this asthmatic murine model suggested that the exacerbation of lung pathology observed in asthmatic mice was not attributed to alternation of viral replication-mediated tissue damage but rather to enhancing lung pathology mediated by synergistic effects of allergic airway inflammation and influenza virus infection on production of some inflammatory mediators. This hypothesis was supported by the findings of severe inflammatory cell infiltration around the airways and upregulation of chemokines such as MCP-1/CCL2, MIP-1α/CCL3, and IP-10/CXCL10 in the lungs of virus-infected asthmatic mice with subsequent induction of allergic airway inflammation. This asthmatic model mice also showed significant weight loss after influenza virus infection while virus-infected mice in which acute allergic airway inflammation was not induced developed less severe weight loss, which is the most important clinical sign of deterioration when assessing the pathogenesis of influenza virus infection in mouse models [35], indicating that influenza virus infection with subsequent allergic airway inflammation also enhances the severity of illness in addition to lung pathology. Furthermore, the virus-infected mice in which acute allergic airway response was subsequently induced also showed stronger suppression of OVA-induced airway eosinophilia than uninfected mice, supporting the findings of a previous report by Wohllenben et al. [33] showing that influenza virus infection inhibits efficient recruitment of Th2 cells to the airways. It is also important to note that our findings contradict some of the findings reported by Wohllenben et al. [33], whose mice did not show any weight loss. There are some major differences between the study of Wohllenben et al. and ours; for example, the former infected C57BL/6 mice with H3N2 virus, while we infected NC/Nga mice with the 2009 pandemic virus. NC/Nga mice are highly sensitive to several types of allergic reactions, including atopic dermatitis and allergic airway inflammation [24, 25]. Thus, the immunopathology of asthma in the presence of influenza comorbidity may differ between NC/Nga and C57BL/6 mice. Our murine model clearly showed exacerbation of lung pathology and clinical signs, mimicking clinical observations in humans [3, 4]. Thus, the murine model described herein may contribute much to our understanding of the pathogenesis of influenza in human asthmatic subjects. Moreover, Wohllenben et al. [33] indicated that influenza virus infection did not influence the development of goblet cell metaplasia, despite suppressing Th2 responses. Airway remodeling is a major contributor to the pathogenesis of asthma, particularly the development of airflow obstruction and the progressive decline in lung function [36]. Airway epithelial cells play an important role in the airway remodeling associated with asthma. Asthmatic patients show an increase in the number of goblet cells, referred to as goblet cell metaplasia, which is not caused by the proliferation of pre-existing goblet cells but is rather attributable to the transdifferentiation of ciliated cells and Clara cells [8]. Goblet cell metaplasia is noted in mild, moderate, and severe forms of asthma, although the finding is much more common in cases of severe and fatal asthma [37, 38]. In contrast to the previous report [33], influenza virus-infected mice with subsequent allergic airway reactions demonstrated a clear increase in goblet cell metaplasia when compared with infected mice without subsequent allergic airway reactions or with uninfected asthmatic mice. However, the mice with prominent goblet cell metaplasia did not show obvious structural changes in subepithelial region that are commonly affected during airway remodeling process of asthma, indicating that the pathogenetic mechanism of goblet cell metaplasia in this model might be different from that of conventional asthmatic airway remodeling, which is genetically distinguishable from acute allergic responses characterized by eosinophilic inflammation [39]. Furthermore, allergen challenge alone (Mock/OVA mice) did not induce goblet cell metaplasia in this model. In previous murine models of asthmatic airway remodeling, mice were challenged with OVA for 6 times to induce airway structural change [40]. Taken together, this observation and pervious repot suggested that OVA challenge alone for two consecutive days in the murine model developed herein was not sufficient to induce conventional asthmatic airway remodeling, and influenza virus infection on the bronchiolar epithelium plays a key role in inducing goblet cell metaplasia in the presence of allergic airway inflammation. It was reported that Influenza virus infection induces bronchial epithelial regeneration with the formation of a nonkeratinizing stratified squamous metaplasia in later stages of infection [41]. Acute allergic airway inflammation might skew the epithelial regeneration process to goblet cell metaplasia form squamous metaplasia in this murine model.

Some recent reports indicate that acutely allergic airways are highly resistant to influenza virus infection [12–14]. The data derived from the influenza virus infectious model with subsequent acute allergic airway inflammation contradict these findings. The temporal relationship between allergic airway inflammation and influenza virus infection reported herein is different from that reported in the previous studies. These previous studies used a model in which OVA-sensitized mice were infected with influenza virus after OVA challenge. Pre-existing allergic airway inflammation affects the immunological status of the lung, leading to mitigation of virological and immunopathological outcomes. Therefore, the susceptibility to influenza virus infection and the nature of the immune responses would be expected to be different between subsequent allergic airway inflammation and pre-existing allergic airway inflammation models. Moreover, the pre-existing allergic airway inflammation model based on NC/Nga mice revealed that pre-existing allergic airway inflammation induced resistance to influenza virus infection, particularly that of the airway epithelium, which plays a role in allergic inflammation; these data support those reported previously [12–14]. Epithelium that was resistant to influenza virus infection also showed less goblet cell metaplasia than that which was not. Treatment with a neuraminidase inhibitor reduced the influenza virus-induced inflammatory response, but did not inhibit infection to the bronchiolar epithelium or subsequent epithelial metaplasia. Taken together, these observations also support the hypothesis that influenza virus infection plays a critical role in inducing goblet cell metaplasia of infected epithelium in the presence of allergic airway responses.

In conclusion, we generated and characterized a novel murine model of asthma and influenza comorbidity and found that influenza virus infection followed by induction of allergic airway responses in asthmatic mice led to an exacerbation of lung pathology including pulmonary inflammation and subsequent epithelial goblet cell metaplasia. These observations were quite consistent with those of clinical and epidemiological studies in asthmatic patients. In contrast, pre-existing allergic airway inflammation protected from subsequent influenza virus infection, which was consistent with the previous comorbidity murine models of influenza and asthma. These two different outcomes of this murine model developed herein suggest that the temporal relation between influenza virus infection and allergic airway inflammation might be one of the determinants for comorbidity of influenza virus infection in asthmatics. Thus, the model developed herein will be useful for investigating the pathogenesis of influenza virus infection in humans with asthma and for improvements of patient care and medical treatments.
